# Myopia: Risk Factors, Disease Mechanisms, Diagnostic Modalities, and Therapeutic Options

**DOI:** 10.1155/2018/7942379

**Published:** 2018-06-26

**Authors:** Malgorzata Mrugacz, Marzena Gajecka, Ewa Mrukwa-Kominek, Katarzyna J. Witkowska

**Affiliations:** ^1^Medical University of Bialystok, 15-089 Białystok, Poland; ^2^Poznan University of Medical Sciences, 61-701 Poznań, Poland; ^3^Medical University of Silesia, 40-055 Katowice, Poland; ^4^Medical University of Vienna, 1090 Wien, Austria

Myopia is a global problem, being particularly prevalent in the urban areas of East and Southeast Asia. The distribution of myopia according to the World Health Organization is not equal in different countries and the age groups. In children, the prevalence of myopia is 11.7% and ranged from 4.9% in Southeast Asia to 18.2% in the Western Pacific Region. In adults, the prevalence of myopia is 26.5% and ranged from 16.2% in the Americas to 32.9% in Southeast Asia. The results of metaregression showed that the prevalence of myopia increased from 10.4% (1993) to 34.2% (2016) [1]. In 2050, a total of 4758 million people worldwide (49.8% of the world's population) are expected to be myopic, and 938 million people (9.8% of the world's population) are expected to suffer from high myopia. In addition to the economic and social burdens, associated ocular complications may lead to visual impairment [2]. Myopia has a diverse etiology, with both environmental and genetic factors believed to be involved in the myopia's development and progression. Genetic linkage studies have mapped the dozen loci, while association studies have found more than 70 different genes. Many of these genes are involved in common biological pathways known to mediate extracellular matrix composition and regulate connective tissue remodelling. Other associated genomic regions suggest novel mechanisms in the etiology of high myopia, such as mitochondrial-mediated cell death and photoreceptor-mediated visual signal transmission. The environmental factors implicated in myopia include near work, light exposure, lack of physical activity, diet, a higher level of education, and urbanization. The interactions between genes and environmental factors may be significant in determining individual risks of high myopia and may help explain the pathogenetic mechanisms of myopia in human population.

In the past years, various techniques had been used to study ocular blood in myopia, such as fluorescein angiography (FA), indocyanine green angiography (ICGA), color Doppler imaging (CDI), optical coherence tomography (OCT), and optical coherence tomography angiography (OCTA). These tools provide a noninvasive and quantitative approach for monitoring choroidal and retinal changes in pathologic myopia. Especially, OCTA is an imaging technique that enables high-speed, high-resolution, and depth-resolved imaging of the retinal and choroidal vasculatures in myopia-related complications diagnosis such as chorioretinal atrophy and choroidal neovascularization (CNV).

Most nearsighted patients observe marked improvement with treatment including corrective lenses, corneal refractive therapy, and refractive surgery. Early treatment of myopia can prevent social and academic difficulties that can accompany poor vision.

The first paper of this special issue addresses the axial length growth depending on the season and the type of behavior and demonstrates the impact of regular sporting activities and day light exposure as preventative factors against the eyeball growth. The second paper presents the study on altered retinal cones/opsins induced by monochromatic lights that might be involved in the refractive development in guinea pigs. The third paper is on the choroidal thickness (CT) and retinal thickness (RT) in highly myopic tessellated eyes and the role of early quantitative assessment of choroidal thickness and qualitative examination of choroid morphology in predicting myopic maculopathy. The fourth paper of this special issue presents nonpharmacological therapeutic possibilities of refraction defect prevention in young adults, with special regard to myofascial therapy, osteopathy, and massage of acupuncture points surrounding the eye. The fifth paper describes the correlations between peripapillary vessel density, retinal nerve fibre layer (RNFL) thickness, and axial length (AL) with optical coherence tomography angiography (OCTA) in myopia. The sixth paper addresses the relationship between simple hemorrhage (SH) associated with lacquer crack (LC) and myopic choroidal neovascularization (CNV) in high myopia. The seventh paper proposes the newly developed diagnostic techniques for the assessment of ocular blood flow in myopics. The two subsequent papers present the prevalence and related factors for myopia in school-aged children. Intensive near work (writing, reading, and working on a computer) leads to a higher prevalence of myopia, watching television does not influence the prevalence of myopia, and being outdoors decreases the prevalence of myopia.

*Malgorzata Mrugacz* *Marzena Gajecka* *Ewa Mrukwa-Kominek* *Katarzyna J. Witkowska*
